# Impact of home-based squat training with two-depths on lower limb muscle parameters and physical functional tests in older adults

**DOI:** 10.1038/s41598-021-86030-7

**Published:** 2021-03-25

**Authors:** Akito Yoshiko, Kohei Watanabe

**Affiliations:** Faculty of Liberal Arts and Sciences, Chukyo University, Toyota, Aichi Japan

**Keywords:** Ageing, Physiology, Health care, Geriatrics, Medical imaging, Quality of life

## Abstract

This study investigated the effect of home-based shallow and deep squat trainings on knee extension peak torque, muscle thickness, one-repetition maximum (1RM) leg press, and physical function in older individuals. Sixteen participants were randomly assigned to the shallow squat group (SS group; age, 71.0  ±  4.0 years) or deep squat group (DS group, age; 68.6  ± 3.6 years). Chairs of 40-cm height and chairs with a cushion of 20-cm height (60-cm in total) were used as the depth targets for squats, with participants instructed to sink until their hip touched the chair and cushion. Participants performed four sets of squats per day (35 repetitions per set), three days per week, for 12 weeks at their home. Knee extension peak torque, muscle thickness of quadriceps femoris (e.g., vastus lateralis, rectus femoris, and vastus intermedius), and physical function were measured at weeks 0 (baseline), 4, 8, and 12. Maximal isometric knee extension peak torque, muscle thickness, and walking speed did not change significantly over the 12-week training period in either group (*P* > 0.05). However, compared with the baseline, there was significant improvement in the results of 30-s sit-to-stand repetition tests after weeks 8 and 12 in both groups (*P* < 0.05). Additionally, 1RM leg press results were significantly improved after weeks 4 and 12 in the DS group, and weeks 4, 8, and 12 in the SS group (*P* < 0.05). Results indicate that home-based weight-bearing squat training improves lower limb function in older adults, as well as performance in physical functional tests related to activities of daily living. Moreover, such training benefits older adults regardless of whether squats are shallow or deep.

## Introduction

Skeletal muscle mass and strength decrease with age^1^. Reduced muscle strength is related to decreased independent living, quality of life, and life prognosis^2^. Resistance exercise for older individuals is considered an ideal intervention for improving and controlling the decline of skeletal muscle mass, strength, and physical dysfunction. Many studies indicate that chronic resistance training increases muscle quantity and improves muscle strength, thereby improving physical function in older adults^3–5^.

Weight-bearing squats are widely performed as part of home-based resistance exercise for thigh muscles—muscles necessary for walking, ascending and descending stairs, and rising from chairs. Home-based weight-bearing squat exercises are easy to understand, safe, and inexpensive^6^. According to Fujita et al.^7^, when performing a weight-bearing quarter squat that flexes the knee joint at a 45° angle from the standing position (0°), the muscle activity of the vastus lateralis (VL) and rectus femoris (RF) in older adults is typically less than 50% of the maximum voluntary contraction (MVC). Interestingly, these muscle activities in older adults were approximately three times higher than those in young adults^7^, suggesting that squat training is relatively stressful for older adults even when performed in the same training with young adults. Previous studies have shown that home-based resistance training, including squat training, significantly increases thigh muscle strength and quantity in older adults^6, 8–10^. For instance, Yoshiko et al.^10^ found that 10 weeks of weight-bearing training, including squats, improved physical function and increased muscle thickness (MT). Based on its proven benefits, weight-bearing squat training is a recommended exercise for older individuals.

Knee joint subduction during squats (i.e., squat depth) has been used as a control factor, with three knee joint subductions used for squat training research, namely, partial, parallel, and full squats^11^. Research also indicates that RF muscle activation at a squat position with a 90° knee joint angle (full extension: 0°) is significantly higher than that with a 20° angle^12^. Several studies show that deep knee bended squat (DS) training as a full or parallel squat produces greater increases in knee extension peak torque than shallow knee bended squat (SS) training as a quarter or partial squat^13–15^. Interestingly, McMahon et al. observe a significant increase in knee extension peak torque at knee joint angles of 110°–130° in SS groups and of 75°–150° in DS groups after several weeks of squat training^14^. These results suggest that the squat training effects on thigh muscle function are closely related to knee joint angle. However, these results were produced under three specific conditions: (1) participants performed barbell squats, not weight-bearing squats; (2) training was strictly supervised by a trainer; and 3) participants comprised younger adults alone. As such, it is unclear whether the results of McMahon et al.’s study are replicable for weight-bearing squat training, home-based training, and among older adults.

Scholars have measured muscle quantity—such as muscle thickness, cross-sectional area, and volume—to determine the effect of squat training because these parameters influence force-generation capacity^16, 17^. In this respect, numerous studies have reported that 10–12 weeks of squat training, either with or without barbells, effectively increases the quantity of knee extension muscles^9, 10, 13, 15^. Recent studies have examined the effects of squat training on the functional and morphological characteristics of individual knee extension muscles^18, 19^. For instance, Kubo et al.^15^ found that VL muscle volume, but not RF muscle volume, was significantly increased by squat training. Investigating acute muscle activation in thigh muscles during unloaded squats, Isear et al.^20^ found that the VL muscle activation was higher than RF muscle activation during the shallow knee bended phase (i.e., when the knee joint angle moves from 30° to 60°) but observed no such difference during the deep knee bended phase (i.e., when the knee joint angle moved from 60° to 90°). Meanwhile, Akima et al.^18^ investigated muscle activation in four knee extensor muscles—namely, the VL, RF, vastus intermedius (VI), and vastus medialis—during a leg press, which is a similar movement to the squat. In doing so, they found that the VI activation level during concentric was significantly lower than that of the VL during the shallow phase but the same during the deep phase. Normalized muscle activation level during movement may reflect the absolute load to the muscles^7, 12, 21^, inferring that the effect of squat training on muscle quantity would differ between the individual muscles of the knee extensor. To avoid missing the training effect on muscle quantity, it may be better to measure training effect in a few parts of the quadriceps femoris (QF). More specifically, it appears likely that an increase in muscle quantity will be greater in the VL than in the RF and VI in an SS training group. However, scholars have yet to investigate the effect of weight-bearing squat training on individual muscles of the QF. Understanding individual synergistic knee extensor muscles may clarify the underlying mechanism of physical function because individual QF muscles significantly influence activities of daily living (ADL) in older individuals^22, 23^.

This study investigated the effect of two depths of home-based weight-bearing squat training on knee extension peak torque, MT, one-repetition maximum (1RM) leg press, and physical function in older individuals. This study specifically examined home-based training for older individuals using equipment available in the home (i.e., a chair and cushion) to determine squat depth. We hypothesized that knee extension function at an extended knee joint angle (e.g., 30°) would increase the effects of both SS and DS squat training, whereas that at a flexed knee joint angle (e.g., 70°) would only see increased effects in DS training. We also hypothesized a larger increase in muscle quantity in the VL than in the RF and VI in both the SS and DS training groups.

## Methods

### Participants

Participants were recruited at a health promotion class held at the Chukyo University. Participants aged 65 years or older who possessed a chair, CD deck, and cushion in their residence were included in the study. Exclusion criteria included dependence on others for ADL; being diagnosed with impairment of cognitive function and/or dementia by a medical doctor; having a musculoskeletal, neuromuscular, orthopedic, and/or cardiovascular disease; and being advised to limit exercise, sports, and physical activities by a medical doctor. As summarized in Table 1, participants comprising 16 older (3 men, 13 women) adults participated in this study. An international activity questionnaire (IPAQ) was used to obtain participants’ baseline physical characteristics and activity level^24^. Stratified semi-randomization was used to assign participants to the SS group (n = 8) and DS group (n = 8); this assignment took into consideration the balance of age, gender, and body mass index (BMI) between groups. All of participants completed this experiment.

**Table 1:** Baseline physical characteristics parameters, daily physical activity, muscle thickness, knee extension peak torques and physical functional tests in shallow squat (SS) group and deep squat (DS) group.

	SS group (n = 8)		DS group (n = 8)		*P* value	ES
**Physical characteristics**						
Age	71.0 ± 4.0		68.6 ± 3.6		0.34	0.23
Gender (men / women)	1 / 7		2 / 6		0.54	0.16
Height (cm)	155.1 ± 7.5		155.3 ± 3.9		0.96	0.03
Body mass (kg)	54.8 ± 9.0		54.1 ± 6.5		0.72	0.11
BMI (kg m^−2^)	22.6 ± 2.1		22.4 ± 2.2		0.88	0.03
Muscle mass (kg)	21.0 ± 3.4		21.0 ± 2.3		0.88	0.08
SMI (kg m^−2^)	8.7 ± 0.7		8.7 ± 0.8		0.72	0.11
Body fat (%)	28.6 ± 3.5		27.8 ± 4.7		0.80	0.05
**Daily physical activity**						
Sitting (min wk^−1^)	1890.0 ± 985.0		2415.0 ± 736.1		0.33	0.27
Total activity (MET min wk^−1^)	1416.6 ± 993.8		978.0 ± 598.0		0.33	0.26
Estimated activity energy expenditure (kcal wk^−1^)	1255.7 ± 770.5		921.6 ± 594.5		0.38	0.24
Physical activity level (Low/ Moderate / High)	1 / 5 / 2		4 / 4 / 0		0.14	0.49
**Muscle thickness**						
RF (cm)	1.57 ± 0.33		1.50 ± 0.33		0.72	0.11
VL (cm)	1.59 ± 0.37		1.49 ± 0.37		0.80	0.08
VI-anterior (cm)	1.44 ± 0.40		1.33 ± 0.35		0.51	0.18
VI-lateral (cm)	1.17 ± 0.32		1.20 ± 0.26		0.80	0.08
**Knee extension peak torque**						
Knee joint angle at 30° (Nm)	63.03 ± 26.15		62.85 ± 13.65		0.72	0.11
Knee joint angle at 70° (Nm)	65.81 ± 19.57		58.87 ± 23.12		0.44	0.21
Knee joint angle at 110° (Nm)	68.67 ± 27.27		57.80 ± 11.95		0.28	0.29
**Physical functional tests**						
Preferred walk speed (m s^−1^)	1.56 ± 0.25		1.57 ± 0.15		0.72	0.22
Fast walk speed (m s^−1^)	2.03 ± 0.27		1.95 ± 0.23		0.38	0.09
1RM of leg press (kg)	51.88 ± 17.30		55.88 ± 19.63		0.51	0.18
Sit-to-stand (reps)	22.38 ± 3.20		22.13 ± 6.45		0.57	0.15

Values are shown as mean ± SD. ES, effect size; BMI, body mass index; SMI, skeletal muscle mass index; MET, metabolic equivalent of task; RF, rectus femoris; VL, vastus lateralis; VI, vastus intermedius; RM, repetition maximum.

Before the experiment, the purpose, procedures, and risks related to this study were explained to each participant, who then provided written informed consent. All examination protocols were approved by the Chukyo University Research Ethics Committee (No. 2018-003). The study was conducted in accordance with the ethical principles stated in the Declaration of Helsinki.

### Experimental procedure

During the first visit, we introduced the purpose and significance of the study to the participants and explained the entire experimental protocol, specific training procedures, and measurement techniques. All participants provided written informed consent before participating in the study.

We then examined participants’ height and other physical characteristics (e.g., body mass, muscle mass, and body fat) using an analog height meter and bioelectrical impedance analysis (InBody 430; InBody Japan, Tokyo, Japan), and surveyed their daily physical activity level. The skeletal muscle mass index (SMI) was calculated as follows: SMI (kg/m^2^) = muscle mass / height^2^. The InBody 430 body fat analyzer measures impedance across the legs, arms, and trunk via multiple frequencies of 5 kHz, 50 kHz, and 250 kHz. The system’s eight electrodes are contained in footpads mounted on the surface of a platform scale and in handheld pads in the handles extending from the machine’s body. Participants were instructed to stand on the electrode plate while squeezing the handle with both hands, and to maintain this posture for 1 or 2 min. The machine automatically measures impedance and body mass, while the participant’s height and age are manually entered into the system. Sitting time, total activity, estimated activity energy expenditure, and physical activity level were calculated using the IPAQ. At same timing, the participants practiced physical functional tests, e.g., MVC during knee extension, 5 m preferred walking test, 5 m fast walking test, 1RM leg press, and sit-to-stand test, in order to control for experience bias.

Based on these results, participants were semi-randomly assigned to the SS or DS exercise group. They performed the prescribed trainings at their home for 12 weeks. All participants were evaluated in the laboratory via ultrasound examination, the MVC of knee extension, and physical functional tests a total of four times at 4-week intervals: namely, weeks 0 (baseline), 4, 8, and 12. Participants were requested to maintain their usual dietary and physical habits during the study period (12 weeks).

### Squat training program

**Figure 1:**
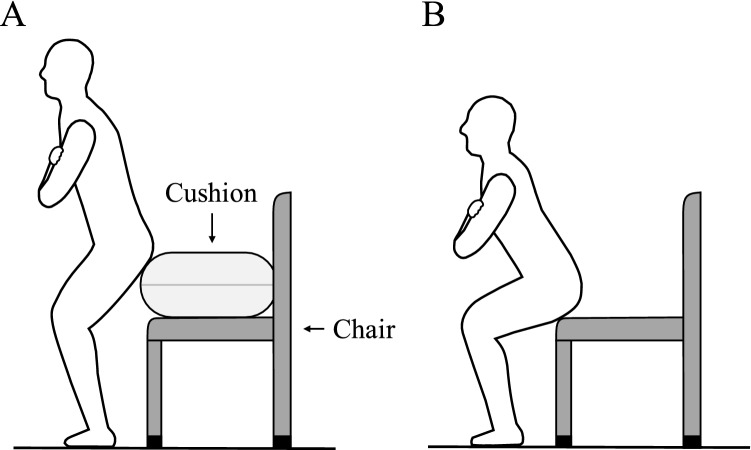
Representaative image of shallow squat (**A**) and deep squat (**B**) using chair and cushion.

This study used two home-based weight-bearing squat training programs—namely, SS and DS training—which managed target depth using a chair and cushion (Fig. 1). Significantly, these programs can be performed at home using household items and do not require any specialized exercise equipment. Participants were instructed on the correct exercise techniques during the first visit and were then able to perform the exercise at home. Participants kept tempo of 55 bpm using a CD.

In the SS group, SS were performed using a chair with a height of 40 cm and a cushion with a height of 20 cm, making up a total height of 60 cm (Fig. 1A). Participants in the SS group were instructed to position themselves with their back to the chair (i.e., as if they were going to sit in the chair) with their feet at shoulder width. SS began at the standing position, with one repetition comprising an upper and lower limb movement for eight beats. The eight-beat SS movement was explained as follows: on the first beat, touch your left shoulder with your right arm; on the second beat, touch your right shoulder with your left arm; on the third and fourth beats, sink until your hip touches the cushion; on the fifth and sixth beats, return to a standing position; on the seventh beat, lower your left arm; and finally, on the eighth beat, lower your right arm.

In the DS group, DS were performed using a 40-cm high chair (Fig. 1B). DS began at the same position as SS, and a repetition similarly comprised an eight-beat pattern. The eight-beat DS movement was explained as follows: on the first beat, touch your shoulders with your right and left arms so that your arms are crossed in front of your chest; from the second to fourth beats, sink until your hip touches the seat of the chair; from the fifth to seventh beats, return to standing position; and, on the eighth beat, lower both arms. In contrast to SS, DS involved a further beat for sitting and standing because DS involves a larger range of motion. Participants were instructed to “do the squat until your hip just touches the cushion or chair, do not actually sit on the cushion or chair” and to “stop the movement when you feel the cushion or chair near your hip, and return to the standing position in accordance with the rhythm.”

Both groups performed four sets of squats a day for 3 days a week (i.e., 12 sets per week) over the 12-week training period. One set comprised 35 repetitions (i.e., 140 repetitions per day). A single uninterrupted set (35 repetitions) performed in accordance with the set tempo took approximately 6 min to complete on an average. Participants were instructed to perform four sets over the course of a day and not in a row; we recommended that they perform two sets in the morning and two sets in the afternoon. We checked participant form and confirmed that were practicing squats correctly every 4 weeks when they came into the laboratory for tests. Participants recorded their training using a customized recording log. They kept a log at home during the training period. We instructed them to note down after the training immediately. They brought the log along with them and showed it when they came to the laboratory every 4 weeks, and we checked it. After the 12-week training program, we collected participant logs and counted how many participants completed the training.

### Ultrasound measurements

As in previous studies^10, 25, 26^, we measured the muscle thickness (MT) of the mid-thigh using an ultrasound device. MT is affected by muscle contraction-induced blood flow and fluid shifts. To avoid these effects, participants were directed to rest on a bed for at least 15 min before taking ultrasound measurements. After the rest period, the participants were moved to an examination bed and placed in the supine position to enable the measuring of their anterior and lateral regions with their knee joints fully extended. We measured the portion of the right thigh corresponding to the midpoint between the greater trochanter and lateral condyle.

The LOGIQ e premium ultrasound system (GE Healthcare, Duluth, GA, USA) was used to obtain images. This real-time B-mode ultrasonographic device has a 3.8 cm width and 8–10 MHz linear array probe for imaging with the following acquisition parameters: frequency, 10 MHz; gain, 35 dB; depth, 4–6 cm; and focus point, 1 (top of the image). Depth was determined according to the individual participant, but generally ≤ 6 cm; the same depth was used in each measurement. We measured images from two sites, namely, the anterior and lateral thigh. The following images were captured: the RF, VI and anterior femur, and the VL, VI, and lateral femur aligned in a straight line^10^. The MT of the RF and VL was defined as the distance between the superior border of the subcutaneous fascia and deep aponeurosis. In the case of the VI, muscle thickness was defined as the distance between the inferior border of the superficial aponeurosis and superior border of the femur. A water-soluble gel was applied to the scanning head of the probe to achieve acoustic coupling, and extra care was taken to avoid deformation of the muscle morphology when placing the probe on the skin surface.

Three frozen images of each section were stored in the digital imaging and communications in medicine format and transferred to a personal computer for later analysis. ImageJ software, version 1.46 (National Institutes of Health, Bethesda, MD, USA), was used for analysis.

### Physical functional tests

Participants performed six functional tests in a laboratory setting: namely, MVC during knee extension (two trials), 5 m preferred walking test, 5 m fast walking test, 1RM leg press, and sit-to-stand tests. Subjects performed MVC during isometric knee extension at knee joint angles of 30°, 70°, and 110° (0° corresponds to full extension) using a dynamometer mounting force transducer (VINE, Tokyo, Japan). As in previous studies^25, 27^, MVC measurement and knee extension peak torque (KEPT) calculation were conducted using the right leg. The hip was fixed to the dynamometer using a strap, with the hip joint at 90° flexion, and the ankle was attached to a pad linked to the force transducer. The knee extension tasks at the three selected knee joint angles were performed in a random order. The MVC trial included a gradual increase in knee extension force to maximum effort in 1–2 s, and a plateau phase at maximum effort was maintained for 4 s. Participants performed at least two trials with a ≥ 2-min rest interval between them. The highest MVC force was selected from the trials. KEPT torque was calculated as the product of the MVC force and distance of the lever arm.

For the 5 m preferred/fast walk test, four parallel lines were taped on the floor at a distance of 1 m, 6 m, and 7 m (finish line) from the start line (0 m). Participants then walked from the starting to the finish line at their usual and maximum speed. The researcher timed how long it took participants to walk from the 1 m to 6 m lines while walking alongside them^10^.

The 1RM leg press was measured using a seated leg press machine, with the participants in a sitting position with the knee bended at a 90° angle. We used right leg for all participants. Participants extended the knee joint angle from 90° to 10° with progressively heavy weights until they could no longer lift the weight; we then determined submaximal weight. After a 5-min break, maximum repetition was determined using the submaximal weight. We adopted this protocol to reduce the risk of injury and hypertension from strain. The 1RM was calculated using the submaximal weight and maximum repetition by referring to the maximum based on the repetitions chart^28^.

Finally, the participants performed as many sit-to-stand as possible for 30 s with their arms crossed in front of their chest. For this test, the height of the seat was 40 cm from the floor.

### Statistical analyses

All values are reported as means and standard deviations. Before the analysis, we performed the Shapiro–Wilk test to assess the normal distribution of data for further analysis. As our results included non-normal distributed data, we used nonparametric statistical tests. The Mann–Whitney test was used to compare physical characteristics (i.e., age, height, body mass, BMI, muscle mass, SMI, and body fat), daily physical activity, MT, and physical functional tests (i.e., KEPT, 5 m preferred/fast walking test, 1RM leg press, and sit-to-stand) between the groups at week 0. Chi-square tests were used to analyze differences between gender and physical activity level. MT and physical functional tests were normalized by using the values from week 0 to test the effect of intervention. The Friedmann test was applied to the normalized MT and physical functional tests of weeks 0, 4, 8, and 12 for each participant group. If significant intervention effects were detected, the Wilcoxon test was used to compare the values at weeks 4, 8, and 12 with the value at week 0. In this scenario, the *P*-value was adjusted to avoid the effect of repeatability (the significance level was set at *P* < 0.0125). Effect size (ES) statistics have been provided where appropriate using *r* (Mann–Whitney test and Wilcoxon test), Cramér’s *V* (Chi-square test), and Kendoll’s *W* (Friedmann test). The significance level was set at *P* < 0.05. All statistical analyses were performed using IBM SPSS Statistics, version 22.0J (IBM Japan, Tokyo, Japan).

## Results

Table 1 presents the baseline physical characteristics parameters, daily physical activity, muscle thickness, knee extension peak torques, and physical functional test results for both groups. There were no significant differences between the SS and DS groups in terms of age, height, body mass, BMI, muscle mass, SMI, body fat, MT, daily physical activities, KEPT, and physical function tests at week 0; *P* > 0.05, ES (*r*) = 0.03–0.29, ES (*V)* = 0.16 (gender) and 0.49 (physical activity level).

**Figure 2:**
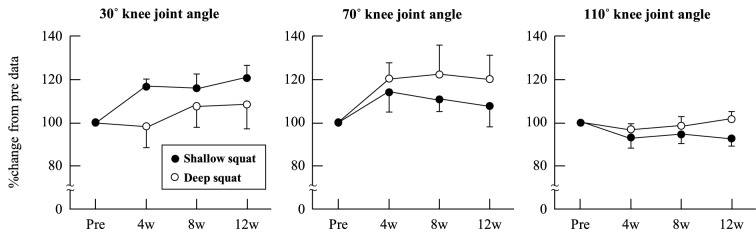
Relative changes in knee extension peak torque at a knee joint angle of 30°, 70°, and 110° as a result of squat training in the shallow squat and deep squat groups. Error bars show standard error (SE).

**Figure 3:**
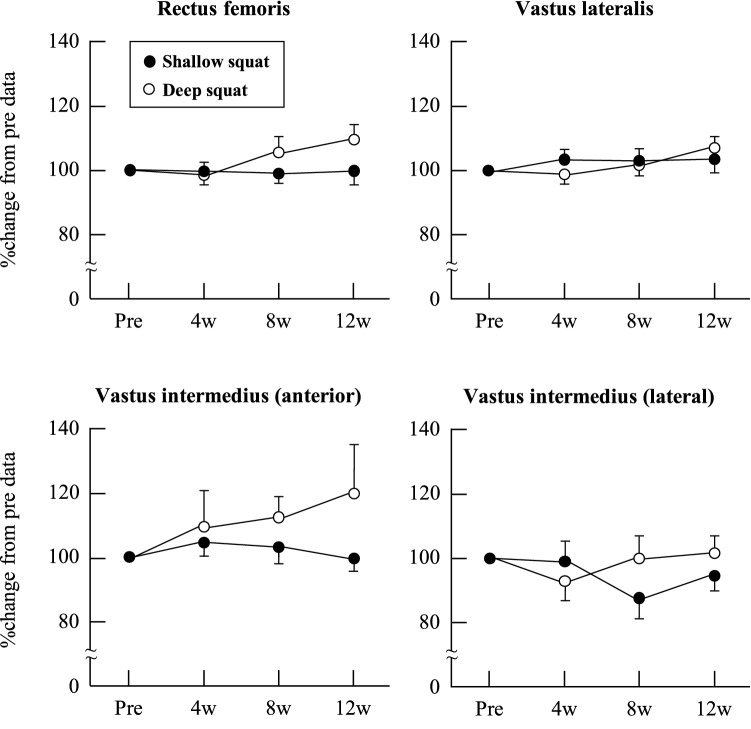
Relative changes in muscle thickness as a result of squat training in the shallow squat and deep squat groups. Error bars show standard error (SE).

**Figure 4:**
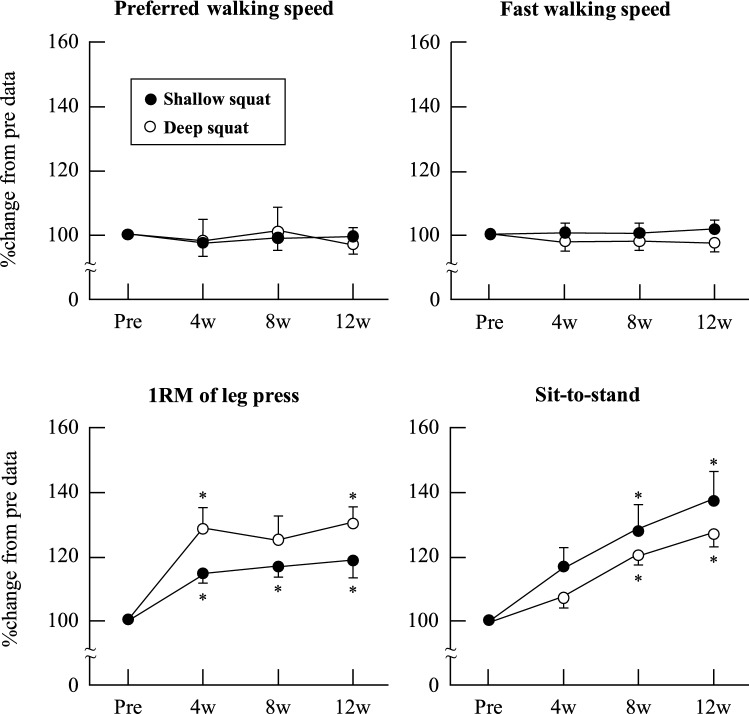
Relative changes in physical functional test results as a consequence of squat training in the shallow squat and deep squat groups. Error bars show standard error (SE). * *P* < 0.05 vs. Pre.

Participants performed their home-based squat training at an average of 12 ± 0 sets per week in the SS group and 12 ± 1 sets per week in the DS group; *P* = 0.89, ES (*r*) = 0.03. KEPT during isometric exercises at knee joint angles of 30°, 70°, and 110° (Fig. 2), MT (Fig. 3), and walking speed (Fig. 4) did not change significantly over the 12-week training period in either group; *P* > 0.05, ES (*W)* = 0.04–0.29. As Fig. 4 shows, sit-to-stand test results showed significant improvement after weeks 8 and 12 in both groups; *P* < 0.05, ES (*r*) = 0.56–0.58. Finally, compared with the baseline (week 0), 1RM leg press results significantly improved after weeks 4 and 12 in the DS group, and weeks 4, 8, and 12 in the SS group; *P* < 0.05, ES (*r*) = 0.56–0.58 (Fig. 4).

## Discussion

This study investigated the effect of two depths (i.e., shallow and deep) of home-based weight-bearing squat training on the KEPT, MT, 1RM leg press, and physical functional test results of older adults. In contrast to our hypothesis, MT and KEPT did not change as a result of the 12-week training regimen in either group. However, sit-to-stand and 1RM leg press performance, influenced by multiple lower muscle activations, significantly improved from week 4 or 8 in both groups. These results indicate that while 12 weeks of consistent training can improve some physical functions in older adults, such training does not impact specific muscle morphology and function. Moreover, home-based squat training had the same overall effect regardless of whether squats were shallow or deep, indicating that squat depth (e.g., partial or parallel) does not impact training intensity in older individuals.

In this study, a primary concern regarding home-based training was whether the participants would complete the training task as directed. We instructed participants to perform 12 sets of squats (35 repetitions per set) at home each week and log their training via a customized recording log. However, some participants did not meet their weekly goal due to bad health or minor pain in their hip and/or knee joint, while several participants exceeded the weekly training regimen by one or two sets. As such, data did not reveal an average of 12 ± 0 sets per week. In contrast, the SS group performed an average of 12 ± 0 sets per week, while the DS group performed an average of 12 ± 1 sets per week. Although participants were reminded to stick to the training schedule during in-person testing every 4 weeks, it was difficult to control the training in this unsupervised home-based training scenario. Nonetheless, results of the Mann–Whitney test (*P* > 0.05, ES (*r*) = 0.03) confirmed that there was no difference in average training sets between the SS and DS groups. Therefore, the following discussion proceeds on the assumption that both groups engaged in the same amount of training.

This study measured KEPT at three knee joint angles: 30°, 70°, and 110°. Noorkõiv et al.^29^ investigated training effects using an isometric knee extension task at the extended knee position (60°) and evaluated MVC force at eight knee joint angles (e.g., 30° − 100°). They subsequently reported that MVC force was significantly increased when the knee was extended (30° − 50°) and did not change when the knee is flexed (60° − 100°) at week 6. In other words, the ability of training to change MVC may depend on the angle of the joints during training. Therefore, we hypothesized that if training was conducted, KEPT may increase at knee joint angles of 30° in the SS group, and 30° and 70° in the DS group. However, in contrast to expectations, the results revealed no significant increase in KEPT in either groups (Fig. 2).

Focusing on older adults, Tsuzuku et al.^9^ found that 12 weeks of weight-bearing training, including squats, significantly increased isometric MVC strength at a knee joint angle of 90°. In Tsuzuku et al.’s^9^ study, participants were instructed to train every day for 12 weeks and increase the repetitions every 4 weeks. In contrast, participants in this study were instructed to train three times a week, with no change in the volume or intensity of training over the 12-week period. These different protocols may explain the inconsistency of training effects. It is also possible that the intensity of weight-bearing squat was insufficient for increasing muscle function in this study’s participants. Using the IPAQ, we measured participants’ daily physical activity, finding that 70% of participants could be categorized as having a moderate or high activity level (Table 1). As participants had a high level of physical activity, the weight-bearing squat regimen may have been relatively low intensity for them. Accordingly, it may be necessary to adjust training frequency and duration according to participant characteristics.

This study sought to observe the change in muscle quantity in individual muscles of the QF because age-related muscle atrophy is not consistent into these synergistic muscles. For instance, RF muscle volume in older adults is 36% lower than that of younger adults, while VI muscle volume is 25% lower in older adults^30^. Evaluating the effect of training on individual muscles of the lower leg may prove invaluable to the recovery or prevention of sarcopenia. In this study, a comparison of MT between week 0 and weeks 4, 8, and 12 of training reveals that training did not have any effect on the MT of the QF in either group (Fig. 3). In other words, results do not confirm muscle hypertrophy after 12 weeks of weight-bearing squat training, regardless of squat depth. Several studies have shown that squat training improves muscle quantity^9, 15^. For instance, Tuzuku et al.^9^ found that the anterior thigh MT of older adults increased after 12 weeks of home-based resistance training. Similarly, Yoshiko et al.^10^ observed an 8% increase of MT in the RF following a ten-week home-based resistance training regimen. In contrast, we observed no change in the MT of the study participants. This discrepancy can be explained by two factors: lower exercise-induced stimulation for the target muscles and this study’s small sample size. With regard to the first factor, Moritani and de Vries^31^ found that the neural contribution to strength gain was higher than that of hypertrophy in older adults, regardless of training duration. This suggests that training-induced muscle hypertrophy requires significant stimulation to specific muscles. Accordingly, it may be the case that the weight-bearing squat intensity of the provided regimen may have been too low for this study’s participants. However, this is a limitation of this training because it is difficult to load the weight over the participant’s body mass. With respect to the second factor, this study had a small sample size of just eight participants per group. Accordingly, the ES was small (SS: ES (*W)* = 0.04–0.11, DS: ES (*W)* = 0.12–0.29) when comparing the MT of participants between weeks 0, 4, 8, and 12. Furthermore, the statistical power of this comparison was 0.06–0.13 (supplemental data). This small power leads to a type II error, indicating its potential of missing the difference. Therefore, the results of this study must be interpreted carefully and further studies involving a larger sample are warranted.

This study found that sit-to-stand test and 1RM leg press results gradually improved over the course of the 12-week squat training regimen. These results support those of studies demonstrating the benefit of such exercises for improving muscle strength in older individuals^8, 32^. Such improvement may be linked to movement dependency, particularly as the movements performed in these tests are similar to those of squat exercises. In this respect, the 30-s sit-to-stand repetition test is frequently used to measure an older individual’s physical function level and to monitor the effects of training^25, 26, 33, 34^. Research shows that multiple muscles are activated during the sit-to-stand and leg press movements, which require movement of the hip and knee joints^35, 36^. McCarthy et al.^34^ found that the sit-to-stand score is correlated (r = 0.33–0.47, *P* < 0.05) with the strength of hip flexion, hip extension, and knee flexion and extension in older adults. As noted, results revealed no change in KEPT (Fig. 2). Hip flexor and extensor muscle function was expected to improve after weight-bearing squat training. Research indicates that the gluteus maximus, which contributes to the movement of hip extension, exhibits higher activation with an increase in squat depth^11^. Similarly, Kubo et al.^15^ found that the volume of the gluteus maximus increased after 10 weeks of squat training. However, we did not measure the change in muscle function and morphology in the hip extensor muscles due to a lack of evidence regarding the validity of hip extensor muscle quantity data produced by ultrasound imaging. As such, it is necessary to conduct further research examining whether the quantity of the hip extensor and flexor muscles increased as a result of our 12-week squat training regimen.

Extant studies indicate that DS squat training has a greater impact on physical function than SS squat training^13, 15^. Indeed, the total amount of work differs between SS and DS training because the latter involves a much larger range of motion. However, we observed very similar changes in the physical functional parameters between squat depths, suggesting no or only negligible differences between the intensity levels of SS and DS. We could not completely capture the total amount of work and intensity of these two squat depths; therefore, it was necessary to understand these parameters from the point of biomechanics and kinesiology. The decrease in daily physical function, such as sit-to-stand and walking ability, is related to sarcopenia^37, 38^, as well as reduced independent living. Considering the improvement in participants’ sit-to-stand test and 1RM leg press results and maintenance of walking speed after the 12-week study period, this study recommends weight-bearing squat training as a means of improving complex functional improvements, irrespective of squat depth/intensity.

### Limitations

This study has three limitations. First, we did not employ a control group as the effect of weight-bearing squat training is aptly demonstrated in the extant research^8–10^. Studies have examined the effects of training on MT, KEPT, walking speed, and sit-to-stand capacity over a 24-week control period, finding no change in MT, KEPT, and walking speed and significant decreases in sit-to-stand capacity^25^. The primary objective of this study was to determine which squat depth (i.e., deep or shallow) is more effective in improving skeletal muscle parameters. While control data may emphasize the effects of the training intervention, this study observed positive changes in 1RM and sit-to-stand test results that clearly demonstrate the benefits of squat training.

Second, this study had a small sample size. Post-hoc ES analysis was conducted as this is an important index supporting the *P*-value. The post hoc ESs of the KEPT, MT, and physical function values were calculated using *W* and *r*, as we used the Friedmann and Wilcoxon tests. The ESs of 1RM leg press and sit-to-stand were large, indicating significant changes (Fig. 4). However, other data were affected by the small sample size. As noted, the statistical power was 0.06–0.13 in MT, 0.06–0.12 in KEPT, and 0.06–0.08 in physical function—with the exception of 1RM and sit-to-stand test results—when comparing data from week 0 and after weeks 4, 8, and 12 (supplemental data). All results should be interpreted cautiously due to the possibility of a type II error. To remove this error, large-scale studies, such as one examining a community-dwelling population, are required in future.

Third, this study did not control for chair and cushion height. Squat depth was generally controlled using knee joint angles or thigh position^14, 29^. Given the difficulty of controlling the angle of squats at the participant’s home, we instructed participants to use a chair with a height of 40 cm and cushion with a height of 20 cm regardless of their physical characteristics (e.g., thigh and calf length). The balance between the height of the chair and length of the lower leg may determine knee joint angle when squatting. Therefore, the angle during the squat movement may vary according to the participant’s body characteristics. Interestingly, Yamada and Demura^39^ found that RF activation during the 40-cm chair sit-to-stand test was similar in subjects regardless of whether they had longer or shorter lower legs. However, it is unclear whether this finding applies to the results of this study. Accordingly, we intend to evaluate muscle activation level under the SS and DS regimens using electromyography in a further study. In this respect, acute response to our squat training protocol needs to be investigated further from a biomechanical and physiological approach.

## Conclusion

This study investigates that the effect of 12 weeks of two depths (i.e., shallow and deep) of home-based squat training on MT, KEPT, and physical function in older adults. We hypothesized that DS would have a greater impact on these parameters than SS. Although neither group exhibited changes in MT and KEPT, both groups showed significant improvement in physical functional parameters, such as sit-to-stand and 1RM leg press capacity, after 4 weeks. Meanwhile, in contrast to expectation, results revealed no discernable difference between the two depths of squat training. These results indicate that squat training may be useful for improving physical functions that resemble the squat movement in older adults and that the overall training effect is not influenced by squat depth/intensity. Results indicate that squat training volume (i.e., frequency and repetition) may be more impactful than the depth of knee bend in squat training.

## Supplementary Information

Supplementary Information
