# Author Correction: Impact of home-based squat training with two-depths on lower limb muscle parameters and physical functional tests in older adults

**DOI:** 10.1038/s41598-021-95096-2

**Published:** 2021-07-29

**Authors:** Akito Yoshiko, Kohei Watanabe

**Affiliations:** Faculty of Liberal Arts and Sciences, Chukyo University, Toyota, Aichi Japan

Correction to: *Scientific Reports* 10.1038/s41598-021-86030-7, published online 25 March 2021

The original version of this Article contained an error in Figure 4, where the y-axis scale for the Preferred walking speed graph was incorrect. The original Figure 4 and accompanying legend appear below.

**Figure 4:**
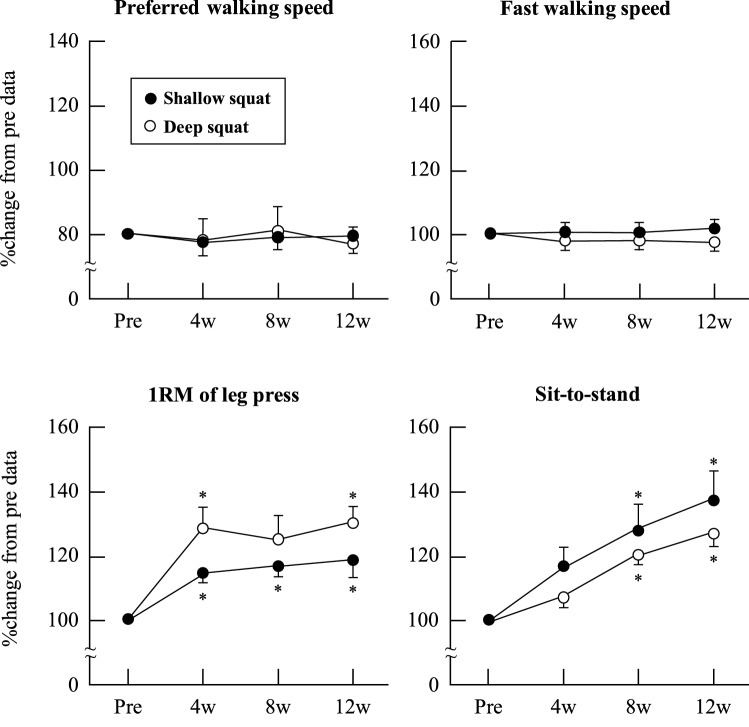
Relative changes in physical functional test results as a consequence of squat training in the shallow squat and deep squat groups. Error bars show standard error (SE). * *P* < 0.05 vs. Pre.

