# Supplementary material for: Impact of home-based squat training with two-depths on lower limb muscle parameters and physical functional tests in older adults
Source: Sci Rep. 2021 Mar 25;11:6855. doi: 10.1038/s41598-021-86030-7 (PMC7994411; doi:10.1038/s41598-021-86030-7)
Supplement: Supplementary file 1 — Supplementary Information [file 41598_2021_86030_MOESM1_ESM.docx]

Research Article

Impact of two-depths of home-based squat training on lower limb muscle parameters and physical functional tests in older adults

Akito Yoshiko^1*^ (yoshiko@lets.chukyo-u.ac.jp)

Kohei Watanabe^1^ (wkohei@lets.chukyo-u.ac.jp)

^1^ School of International Liberal Studies, Chukyo University, Toyota, Japan

^*^Corresponding author: Akito Yoshiko, Ph.D.

School of International Liberal Studies, Chukyo University

101 Tokodachi, Kaizu, Toyota, Aichi 470-0393, Japan

Phone: +81 (565) 46-6952

E-mail: yoshiko@lets.chukyo-u.ac.jp

| Supplemental data. *P* value, effect size and statistical power of the Friedmann test. | | | | | | | | |
| --- | --- | --- | --- | --- | --- | --- | --- | --- |
|  | | Shallow squat group | | |  | Deep squat group | | |
|  | | *P* value | Effect size  (Kendoll’s *W*) | Power |  | *P* value | Effect size  (Kendoll’s *W*) | Power |
| Muscle thickness | |  |  |  |  |  |  |  |
|  | RF | 0.65 | 0.07 | 0.06 |  | 0.14 | 0.23 | 0.11 |
|  | VL | 0.49 | 0.10 | 0.07 |  | 0.07 | 0.29 | 0.13 |
|  | VI-anterior | 0.83 | 0.04 | 0.06 |  | 0.29 | 0.16 | 0.09 |
|  | VI-lateral | 0.47 | 0.11 | 0.07 |  | 0.42 | 0.12 | 0.08 |
| Knee extension peak torque | |  |  |  |  |  |  |  |
|  | Knee joint angle at 30˚ | 0.14 | 0.23 | 0.11 |  | 0.30 | 0.15 | 0.09 |
|  | Knee joint angle at 70˚ | 0.75 | 0.05 | 0.06 |  | 0.13 | 0.24 | 0.12 |
|  | Knee joint angle at 110˚ | 0.20 | 0.19 | 0.10 |  | 0.71 | 0.06 | 0.07 |
| Physical functional tests | |  |  |  |  |  |  |  |
|  | Preferred walk speed | 0.72 | 0.06 | 0.06 |  | 0.58 | 0.08 | 0.07 |
|  | Fast walk speed | 0.79 | 0.04 | 0.06 |  | 0.42 | 0.12 | 0.08 |
|  | 1RM of leg press | < 0.01* | 0.52 | 0.25 |  | < 0.01* | 0.56 | 0.27 |
|  | Sit-to-stand | < 0.01* | 0.91 | 0.52 |  | < 0.01* | 0.73 | 0.39 |
| These data are results of Friedman test. * Significantly difference and proceeding post-hoc test (Wilcoxon test).  RF, rectus femoris; VL, vastus lateralis; VI, vastus intermedius; RM, repetition maximum. | | | | | | | | |
